# “Pets Negotiable”: How Do the Perspectives of Landlords and Property Managers Compare with Those of Younger Tenants with Dogs?

**DOI:** 10.3390/ani8030032

**Published:** 2018-02-27

**Authors:** Taryn M. Graham, Katrina J. Milaney, Cindy L. Adams, Melanie J. Rock

**Affiliations:** 1 Department of Community Health Sciences, Cumming School of Medicine, University of Calgary, 3rd Floor, TRW Building, 3280 Hospital Drive NW, Calgary, AB T2N 4Z6, Canada; katrina.milaney@ucalgary.ca (K.J.M.); cadams@ucalgary.ca (C.L.A.); mrock@ucalgary.ca (M.J.R.); 2 O’Brien Institute for Public Health, Cumming School of Medicine, University of Calgary, 3rd Floor, TRW Building, 3280 Hospital Drive NW, Calgary, AB T2N 4Z6, Canada; 3 Department of Veterinary Clinical & Diagnostic Sciences, Faculty of Veterinary Medicine, University of Calgary, 3rd Floor, TRW Building, 3280 Hospital Drive NW, Calgary, AB T2N 4Z6, Canada; 4 Department of Ecosystem & Public Health, Faculty of Veterinary Medicine, University of Calgary, 3rd Floor, TRW Building, 3280 Hospital Drive NW, Calgary, AB T2N 4Z6, Canada

**Keywords:** pets, dogs, rental housing, tenants, younger adults, moving, animal relinquishment, animal welfare

## Abstract

**Simple Summary:**

In rental housing policy, pets are rarely considered as valued household members. Instead, landlords and property managers are often permitted to ban pets outright, or to advertise them as merely negotiable in their listings for rental housing. In fact, previous research has shown that moving and renting are key reasons for animal relinquishment. To reduce the number of animals that are given up each year due to housing issues, we surveyed landlords and property managers about their perspectives towards pets. Also, because younger adults are disproportionately tenants and because dogs are often banned from rental housing, we interviewed younger tenants with dogs about their recent experiences in the rental market. Our results confirm that dog owners face difficulties in finding rental housing. To keep their pets, tenants made compromises on where and how they lived, which held consequences for their health and that of their pets. Suggestions for improvement are provided, as are implications for research, policy, and practice.

**Abstract:**

Previous research has shown that housing insecurity contributes to animal relinquishment and that tenants with dogs face disadvantages in the rental market. Still, little is known about how dog owners navigate rental markets, nor how landlords and property managers perceive dogs and other pets. This case study reports on in-depth interviews with younger tenants with dogs and on open-ended survey responses from landlords and property managers. In their housing searches, tenants with dogs reported feeling powerless in negotiations and feeling discriminated against. They described settling for substandard properties, often located in less desirable neighborhoods. Also, some said they felt obliged to stay put in these rentals, given how difficult it had been to find a place that would accommodate their dogs. Meanwhile, landlords and property managers indicated that listings advertised as “pet-friendly” tend to receive more applicants than listings in which pets are prohibited. Suggestions for improvement included meeting pets prior to signing the lease; getting everything in writing; steering clear from furnished units; charging utilities to tenants; and speeding up the pet approval process when dealing with condominium boards. These suggestions offer implications for future research, partnerships, and policy options to improve the prospects of pets and their people in rental housing.

## 1. Introduction

Since the early 1980s, research has linked positive health outcomes with pet ownership [1]. More recently, research has linked dog walking with regular physical activity, as well as with positive interactions with neighbors, acquaintances, and even strangers in public spaces [2]. Nonetheless, few studies have inquired into housing status as a factor in people’s capacities to realize such benefits. For instance, giving up a pet to secure rental housing could have an adverse impact on people’s overall health. 

And yet, housing issues, such as moving and renting, remain among the main reasons why people give up their pets [3]. The number of animals that are relinquished due to housing issues is especially problematic during peak moving seasons [4], tight rental market conditions [5,6], foreclosure crises [7], and/or following natural disasters [8]. Then, due to shelter overcapacity, relinquished animals may be euthanized. Euthanasia can have negative impacts on the mental health of staff in shelters [9]. Also, euthanasia based on factors other than the animal’s behavior or health is related to higher employee turnover in shelters [10]. This study calls for a better understanding of how housing status may influence people’s capacities to keep and care for their pets, as an upstream approach to preventing animal relinquishment and the negative ripple effects therefrom.

More than a decade ago, researchers in the US sought to characterize rental housing options for pet owners in their country [11]. They surveyed both tenants and landlords, as well as examined rental listings in newspapers and on websites. Their findings led them to conclude that pets were welcome in fewer than 10% of rental units, whereas about half of the tenants had or wanted pets. Landlords reported charging higher rents and deposits for pet ownership. Tenants with pets reported staying longer in their rentals, compared to tenants without pets. Those with dogs had the hardest time trying to secure a rental. 

More recently, in-depth interviews and an online survey conducted in Sydney, Australia linked supply-demand asymmetry to housing insecurity among tenants with pets [5]. Desperation to find rental housing led some tenants, particularly younger adults, to keep pets without permission. In doing so, these tenants put themselves at risk for eviction and their pets at risk for relinquishment. All tenants with pets, regardless of their age, appeared to be at a disadvantage in the rental market, leading them to rent properties that they otherwise would have refused, thus driving down their overall standard of living. Again, dogs were especially hard to house. 

Building on these insights, we set out to delve into the experiences of younger tenants with dogs. We elected to focus on younger adults, given that they are disproportionately tenants, to the extent of being called “generation rent” [12]. Furthermore, to put these experiences into context, we wanted to consider the perspectives of landlords and property managers. This case study [13] took place in Calgary, Alberta, Canada, which is home to approximately 1.2 million people, 135,000 dogs, and 70,000 cats [14]. Calgary has a reputation for being “pet friendly” [15]; for instance, the city does not limit the number of pets, nor the types of dogs a person can keep [16]. Nonetheless, animal control policies are largely handled at the municipal level in Canada, whereas housing legislation is mainly under provincial jurisdiction. In the Canadian province of Alberta, where Calgary is located, housing legislation permits landlords and property managers to ban pets from rentals, to restrict the number and types of pets that can be kept, and to impose “reasonable” surcharges, such as pet fees or pet rents [17].

## 2. Materials and Methods 

This case study received ethical clearance (REB16-0039) by the Conjoint Health Research Ethics Board at the University of Calgary. All those who participated provided written consent.

### 2.1. Recruitment/Sampling

#### 2.1.1. In-Depth Interviews with Tenants with Dogs 

During the summer of 2016, a purposive sample of younger tenants with dogs was recruited through announcements circulated via social media (i.e., Facebook and Twitter) and through posters displayed on bulletin boards at university and college campuses, as well as at and near off-leash parks. To participate, interested participants needed to live in Calgary; be aged between 18 and 34; consider themselves a dog owner; and have sought rental housing in the past 5 years. The interviews, which lasted approximately one hour each, took place at neighborhood coffee shops or in meeting rooms booked in advance within libraries. The interviews began by asking participants about their experiences with caring for their dogs and proceeded into questions about what it was like to be a pet owner renting in Calgary. Based on digital audio-recordings, each interview was transcribed verbatim in a naturalistic style. To acknowledge their contribution to this study, each participant received a $25 voucher (‘gift card’).

#### 2.1.2. Online Survey of Landlords and Property Managers Who Have Rented to Pet Owners

During the fall of 2016, through social media (i.e., Facebook and Twitter) and with the help of the University of Calgary’s Westman Centre for Real Estate Studies, a convenience sample of landlords and property managers was recruited. Interested respondents were instructed to open a link to the online survey, which included a consent form as a mandatory precursor to participation. The survey began by asking respondents to specify whether they were landlords or property managers. Then, respondents were asked about how many units they rent out and to disclose how many of these they advertise as “pet-friendly.” Building upon a previously administered survey of landlords [11], respondents were next asked about perceived length of stay (i.e., “Do tenants with pets tend to [stay longer/stay about the same/not stay as long] as tenants without pets?”) and about perceived availability of pet-friendly housing in the city (i.e., “Do pet-friendly units tend to receive [more/about the same/less] applicants than units which prohibit pets?”). Following this, respondents were encouraged to share both positive and negative stories about their experiences with renting to pet owners. They were also invited to offer advice to landlords and property managers who may be thinking about renting to pet owners, as well as to prospective tenants who may be searching for housing for themselves and their pets. The survey ended with demographic questions.

We piloted the survey prior to formal data collection and made revisions according to feedback received. Specifically, the open-ended questions did not change, yet we removed the demographic questions at the end of the survey since piloted respondents advised that some landlords and property managers may not wish to disclose personal information. The final version of the survey ended with two separate questions regarding whether respondents themselves owned pets and whether they lived in the same building or home as their tenants with pets. The survey took approximately 15 min to complete and allowed only for one response per Internet Protocol address. To incentivize participation, the email addresses of respondents who completed the survey were entered into a draw, in which they had a 1 in 50 chance to win a $50 voucher (‘gift card’).

### 2.2. Data Analysis 

Data analysis involved qualitative techniques. To begin, each interview transcript was reviewed line-by-line [18] for key themes related to dog owners’ experiences in the rental market. Next, to put these experiences into context, open-ended survey responses, as provided by landlords and property managers, were reviewed line-by-line. After perspectives from both sides of the rental market were identified and discussed within the research team, the interview transcripts and open-ended survey responses were examined once more to bring together suggestions for improvement.

## 3. Results

In what follows, we begin by describing the participants of our study. Next, we show tenant perspectives related to their recent experiences in the rental market. We then bring in open-ended survey responses from landlords and property managers regarding their experiences with renting to pet owners. Our results end by presenting suggestions for improvement, as provided by landlords and property managers, and as supported by tenants. For simplicity’s sake, we will abbreviate tenants to T, landlords to L, and property managers to PM, when presenting their perspectives.

### 3.1. Participants

A total of 28 dog owners, aged 21 to 31, were interviewed for this study. Each of these participants had recently moved into rental housing within Calgary. Twenty identified as women and eight as men. Twenty-one were born in Canada and the remaining self-identified as Brazilian, Irish, French, Mexican, Polish, Russian, and Vietnamese. Eight were single, 6 in a relationship, 4 common-law, 4 engaged to be married, 2 married, 2 “it’s complicated”, and 2 divorced. Two were pursuing graduate degrees, 12 held bachelor’s degrees, 4 specialized in a trade, and 10 had completed high school. Sixteen kept one dog, 5 kept two dogs, and the remaining kept a combination of dogs and cats. 

Complementing these interviews, we received completed survey responses from 24 landlords and 6 property managers, all of whom were pet owners themselves. The number of units rented out by landlords and property managers varied substantially, from one landlord renting out only a basement unit in their own home to a property manager who was responsible for 382 apartment units.

### 3.2. Tenant Perspectives

**Figure 1:**
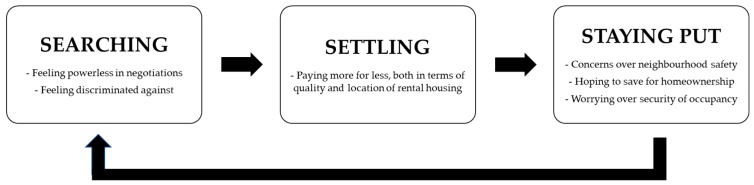
The cycle of rental insecurity for younger tenants with dogs.

Younger tenants with dogs found themselves stuck in a cycle of rental insecurity. This cycle consisted of three stages that lead into one another, namely: searching, settling, and staying put (Figure 1). 

#### 3.2.1. Searching

In their searches for rental housing, younger adults felt powerless in negotiations. T12 (aged 21) was reluctant to look at properties where pets were advertised as negotiable since she thought that “no one would want us as tenants” when indicating, “‘Hey! We’re a young couple! And it’s our first-time renting!’…And then, to be like, ‘We have a dog too!’ Ya. Not good.” Likewise, T9 (aged 25) “looked at some places that said, ‘pets negotiable’ but more often than not, those ones weren’t really negotiable.” She continued, “When we’d visited saying we have two dogs, they were like, ‘Oh, well we were kind of really hoping someone had just one cat.’ So that was a bit tricky…” As T3 (aged 22) explained, trying to negotiate acceptance of a dog “really puts you at a disadvantage, like price-wise and stuff.”

In addition to feeling powerless in negotiations, younger adults believed that landlords and property managers discriminated against them, especially if caring for large dogs or dogs of certain breeds. As T25 (aged 21) recounted, searching for a rental was: “difficult, especially since it looks bad on paper to be two students with two big dogs.” 

In fact, the perception that large dogs were the hardest to house prevailed to such an extent that some tenants opted for smaller-sized dogs, specifically because they knew they would be renting. T3 (aged 22) spoke about why she chose a smaller-sized dog: “Lots of places seem to restrict on size. So that was really important. Like knowing he wasn’t going to be bigger than 20–25 pounds.” T12 (aged 21) shared similar concerns over restrictions placed on dogs in rental housing claiming: “We are lucky since our dog is under the weight limit that is usually seen.” T4 (aged 24) chose a French Bulldog because: “I was living in an apartment at the time and I heard that they were really good dogs for apartments.” As T20 (aged 20) recalled, “breed was a big [restriction]. I’m lucky because my dogs aren’t bully breeds. My friend has two Pit Bulls and couldn’t find a rental property so he’s back living with his dad now. Brutal.” She continued, “Actually, every place I’ve rented with my dogs has expected that I either supply pictures of my dogs or bring [my dogs] so they could meet them…a landlord said to me once, ‘I want to make sure it is an okay breed’. Whatever that means.” T27 (aged 29) also shared, “I was calling one place after seeing it online and the landlord said, ‘Oh, Boxer? No. We don’t take bully breeds…’”

#### 3.2.2. Settling

Where properties did accept pets, younger adults spoke to the substandard quality of these rentals in comparison to the entire pool of available listings. T4 (aged 24) “struggled to find a place that wasn’t run down.” The only decent rental she could find was located on the outskirts of the city, where she had to pay a $750 non-refundable pet fee to keep her French Bulldog, in addition to paying a security deposit equaling one month’s rent. She recounted:

If we do the walk-through and there’s no damage whatsoever at the end, then what’s the purpose of it? The landlord seemed to be expecting damage. That, or just exploiting the fact that there are not many dog places on the market…In the end, I felt cornered to pay because I had no other options.

T2 (aged 30) also found that rental properties which allowed pets were mostly “in horrible shape” and often “more expensive.” To secure a rental for herself and her two smaller-sized dogs, she had to pay $500 in non-refundable fees ($250 per dog), in addition to paying the security deposit. Meanwhile, T15 (aged 29) must have visited “a dozen, if not more” properties, commenting that:

We went to this one basement that allowed dogs and the landlord just invited everyone at the same time. There were like 25 people in this little basement looking at it. And he’s like, ‘Okay, start bidding. Whoever places the highest bid, gets the place.’ And I was like, ‘This place sucks. I’m out.’ Everyone else was bidding! I was like, ‘This place is horrible’…It just didn’t make any sense.

She and her partner finally settled for a unit, yet not without having to pay $500 in non-refundable pet fees ($250 per dog), in addition to paying the security deposit. 

T27 (aged 29) did not face any pet-related surcharges, however, his rental was inaccurately advertised as a duplex, when in fact it was a split-level shared accommodation:

I think the reason why they didn’t ask for extra pet fees was just because of the inconvenient living situation. We’ve asked the landlords to separate the floors. The tenants downstairs have asked the landlords to separate the floors. They even asked for like waterlines so they could have their own sink in their like mini kitchen. The landlords won’t do anything [about the unit] but [they will] allow the dogs. So we’ll deal with it.

Speaking about settling, T17 (aged 28) had to pay $200 extra per month ($100 per dog), in addition to paying the security deposit and $450 in non-refundable pet fees ($225 per dog). She continued, “I had no stove. I had a hot plate. So, I basically ate out every day. I lived there for almost 2 years.”

Simply put, to keep their dogs, younger adults had to make compromises over the quality of their rentals. Many reported paying surcharges, such as non-refundable pet fees and/or monthly pet rents, as part of their costs for settling on a place.

#### 3.2.3. Staying Put

Rental properties that allowed pets were not only perceived to be of poorer quality, they were also typically located in less desirable neighborhoods. The neighborhood where T15’s (aged 29) rental was located was not where she originally hoped to live. She described some of the downsides of her neighborhood, drawing on public safety concerns: “We do not really walk around here…There are a lot of speeding cars. There have been accidents right near our house. Some crazy stuff happens, especially when it gets late.” Instead of walking her dogs outside of her rental, this tenant would drive every day to different off-leash areas throughout the city. In the following months, she and her partner hoped to buy a home, to avoid the difficult process of searching for another rental that would accept their dogs.

T17 (aged 28) was also planning on staying put until she and her partner could ideally save for homeownership. In her experience:

Because I have a Pit Bull, I’m labelled. So our next step is to buy. We will not leave this house and rent again. Because I’m not going to go through what we went through for 7 months trying to find this rental. Therefore, until we can buy, we are going to stay here.

Even when staying put, dog owners reported worrying over security of occupancy. T8 (aged 29) felt nervous when other tenants would comment on her dog’s size: “In the elevator, people would say: ‘Oh, they’re accepting big dogs now?’ And I would get nervous that the property manager was going to later blame me for other people wanting to bring in big dogs.” T12 (aged 21) also lived in fear of receiving complaints, albeit due to her dog’s separation anxiety:

We’re always worried to come back to a noise complaint or something but no complaints yet. That’s why we have that webcam. Just so we know. If someone says, ‘Oh, your dog barked all this time,’ we can be like, ‘Actually he didn’t. We have him on film.’

All in all, younger adults had trouble finding a rental that would accommodate their dogs. Many hoped to avoid searching for another rental for as long as possible, even though staying put sometimes meant fearing for their safety and/or worrying over eviction. 

### 3.3. Landlord and Property Manager Perspectives

Most of the landlords and property managers believed that pet owners tend to stay longer in their rentals as compared to tenants without pets. L10 attributed this longer length of tenancy to the limited options that pet owners have in securing a rental: “I’ve had wonderful long-term tenants with pets. They’re responsible people who do not have many options to move because of their dogs.” Overall, landlords and property managers seemed aware of the difficulties that dog owners face in trying to find rental housing, noting that listings advertised as “pet-friendly” tend to receive more applicants than listings in which pets are prohibited.

As mentioned above, all the landlords and property managers who responded to our survey were pet owners themselves. Thus, they appeared to recognize the importance of keeping pets and their people together. PM7 saw pets as part of the family and realized that having to give up a dog to secure housing would be devastating: “We’ve found that tenants with pets appreciate having a home that welcomes their whole family. We have always had pets; this is one reason we always rent to pet owners.” 

Some landlords and property managers believed that renting to pet owners helped facilitate interactions. L8, who rented out a basement suite, remarked: “My daughters love to interact with our tenant’s pet!” L16 indicated: “When our tenants were moving out, a neighbor came by to tell them just how well-behaved their dog had been.” 

Other landlords and property managers believed that renting to dog owners provided companionship for their own pets. L7, who rents out a basement suite in their house, noted: “I have a dog already so renting to another dog owner provides company for her.” This situation was problematic, however, if the landlord’s dog did not get along well with other animals. L19, for instance, who also rents out a basement suite, found that navigating “yard space is an issue, because we have a dog of our own.” 

Indeed, some landlords and property managers faced difficulties when renting to pet owners. At times, pets had been kept without their permission. When this occurred, L21 “let them keep the pet after meeting the dog and seeing the state of the unit.” By contrast, PM3 “sent a letter saying that they broke what the lease terms were and that they must remove the animals or else be faced with an eviction.” Another instance where tenants needed to be evicted due to pet-related issues arose because neighbors complained about barking dogs who had been “left alone for long hours” (L12). L16 noted that tenants should not “expect to rent condos or shared type accommodations if [they] have a barking dog—[they should] be prepared to rent a house instead.” L6 complained about one instance where a tenant’s cat had sprayed, suggesting that tenants with pets “deal with stains or other issues quickly to avoid lasting damage.” Finally, one landlord had to deal with the temporary rehoming of a pet, after Calgary had been hit by a natural disaster. Specifically, L10 “took in a tenant’s dog after the 2013 flood” so that the tenant could evacuate without worry and so that repairs could get done.

### 3.4. Bringing Together Suggestions for Improvement

Suggestions for improvement included meeting pets prior to signing the lease; getting everything in writing; steering clear from furnished units; charging utilities to tenants; and speeding up the pet approval process when dealing with condominium boards.

#### 3.4.1. Meeting Pets Prior to Signing the Lease 

Before signing the lease, L5 recommended: “meet[ing] the pets first. If [prospective tenants] cannot take care of their pet (super long nails, unkempt coat, or otherwise unhealthy looking), chances are they don’t care about their pet very much and will not care about your rental either.” Similarly, L27 suggested evaluating the commitment a prospective tenant has to ensuring that their pet succeeds: “Meet the people with their pet to ensure the bond is there. Analyze how they interact with their pet. Do they take the time to work with the pet to have it properly trained?” Indeed, as L11 admitted: “Our tenant in our condo has a Doberman that we were somewhat hesitant about renting to because of the breed, but after meeting her and seeing that she is well trained, we weren’t worried anymore.” L16 served as an advocate for larger dogs, suggesting that other landlords and property managers: “Be flexible. Sometimes larger dogs can be much quieter and better for condo living than smaller dogs.” To avoid any potential problems, this landlord recommended: “get[ting] references you can rely on and mak[ing] sure you meet the pet.” Finally, L10 suggested: “meet[ing] the tenant’s pet beforehand and if you have a pet that would be in direct contact with them, let them also meet to ensure it won’t be a problem.” 

Tenants appeared to appreciate when landlords and property managers wished to meet their pets. For instance, T25 (aged 21) suggested that landlords and property managers:

Meet the dog first and see how the potential renter interacts with their dog. I think it says a lot about how the renter is also going to take care of everything they own. And maybe, if they want to see how the animal is in the unit, bring them in there, if they can. And from there, they can decide.

#### 3.4.2. Getting Everything in Writing

Landlords and property managers recommended getting everything in writing, such as limiting the number of pets allowed per unit. When L6 failed to impose a pet limit:

Within one month, their one dog became three dogs, a cat, an iguana, a snake, and a tank of piranhas! In a tiny 500 square foot house with three people in it. And the dogs caused a lot of issues with neighbors as they were not well trained, nor properly supervised.

Following this experience, this landlord recommended that other landlords and property managers: “Be clear in the terms of the lease about the pets, including how many are allowed.” L23 also suggested “get[ting] everything in writing about types of pets, numbers of pets, how nuisances and damages from pets are to be dealt with, etc.” Tenants seemed to respect this practice, when landlords and property managers imposed reasonable pet-related rules. For instance, T4 (aged 24) wanted another dog yet recognized that she could not get one at present, given the terms of her lease, while T12 (aged 21) reported following the rules of her building, including “where dogs need to be on leash and where dogs can use the bathroom.”

#### 3.4.3. Steering Clear from Furnished Units and Charging Utilities to Tenants

To keep their rentals profitable, landlords and property managers suggested steering clear from furnished units, and some also recommended charging utilities to tenants. As L5 revealed:

Our suite is fully-furnished. Our first tenant, who was a friend, rented from us and ended up bringing two cats and one dog. The dog was very sweet but not well trained and the owner let it on all the furniture…We also had problems with utilities being crazy high (utilities are included in our rent) because the owner would go out at night and leave all the lights and TV on for the animals.

Tenants recognized that pets could cause damage to furniture, particularly when it came to puppies that were teething or dogs with separation anxiety who had been left alone uncrated or unconfined. 

#### 3.4.4. Speeding up the Pet Approval Process when Dealing with Condominium Boards 

Depending on the types of properties they rented out, landlords and property managers could potentially lose income in trying to accommodate tenants with dogs. As relayed by L3:

All pets technically must be approved by the condo board, but cats are almost automatically approved, whereas dogs are not…You cannot rent to a dog owner until their dog is approved, which really challenges your rental timeline. For example, if your [previous] tenants give you one month’s notice, then you have one month to advertise and find new appropriate tenants. If their tenancy is dependent upon approval of their dog, the process may take longer than the month, and if they are not approved, you’ve potentially lost one month of rental income. So, I’m inclined to not rent to dog owners, not because I want to exclude them, but merely because of the long process of getting them approved in time without losing rental income, because every month the unit is empty, I pay for everything.

Tenants agreed that getting their pets approved was a slow and stressful process.

## 4. Discussion

The significance of our study lies in its potential to promote housing security for people with pets and to reduce animal relinquishment, given that housing issues remain among the main reasons why people give up their pets [3]. This qualitative study was designed to generate insights and additional questions, not to be representative of all younger tenants with dogs, nor all landlords and property managers. 

Overall, landlords and property managers appeared reluctant to respond to our survey and among those that did participate, all were pet owners themselves. We had not anticipated this result. Previous research has shown that the greatest limitation on the supply of rental housing for people with pets tends to be landlords’ concerns over pet-related damage and nuisance, and that these concerns prevail even among those who have never had actual prior experience renting to pet owners [11]. All the landlords and property managers who participated in our study had actual prior experience renting to pet owners. They seemed aware of the difficulties that dog owners face in trying to find rental housing, noting that housing advertised as “pet-friendly” tends to receive more applicants than units in which pets are prohibited. 

By opening their properties up to pet owners, landlords and property managers can access a wider pool of prospective tenants. Yet at the same time, receiving more applicants can be time-consuming, especially if proper supports are not in place to help alleviate any concerns that landlords or property managers may have regarding pets in rental housing. Meanwhile, depending on the types of properties they rent out, landlords and property managers could potentially lose income in trying to accommodate tenants with dogs. For instance, those in our study who rented out condominium units had to get approval from their board before being able to accept tenants with pets. This pet approval process was described as lengthy and inconvenient, by landlords and property managers as well as by tenants with dogs.

Then again, previous research argues that renting to people with pets may save landlords and property managers costs associated with unit turnover, since pet owners tend to stay longer in rental housing compared to non-pet owners [11]. In our study, most of the landlords and property managers agreed that tenants with pets tend to stay longer in their rentals as compared to tenants without pets. This finding is troubling, however, since tenants in our study also appeared to be paying more for less, both in terms of quality and location of rental housing. 

In their searches for rental housing, younger adults felt powerless in negotiations and felt discriminated against, especially if caring for large dogs or dogs of certain breeds. Where properties did accept pets, many spoke to the substandard quality of these rentals in comparison to the entire pool of available listings. Also, many reported paying non-refundable pet fees and/or monthly pet rents, as part of their costs for settling on a place. These findings are consistent with previous research, which shows that rentals advertised as “pet-friendly” tend to be of poorer-quality [5] and tend to be subject to higher rents and fees [11]. Still, tenants in our study reported staying put in these rentals, given how difficult it had been to find a place that would accommodate their dogs. These rentals were not only of poorer quality, they were often located in less desirable neighborhoods than where younger adults could have lived without a dog.

Understanding the living circumstances of pet owners is important since inequitable access to rental housing may displace some people to locations that limit their capacities to care for themselves and for others. Indeed, research shows that neighborhoods can either provide opportunities for people to engage in health promoting practices or they can constrain people’s capacities to do so [19]. Housing that economically vulnerable people can afford, for instance, is “more likely to be located near noise, pollution, and noxious social conditions” [20] (p. 730). These general principles extend to tenants with dogs who may be forced to live in less desirable neighborhoods, thus limiting their capacities for physical activity (i.e., via dog walking) and for community development (i.e., via dog-facilitated positive interactions). 

In our study, some younger adults reported feeling unsafe walking their dogs outside of their rentals, which added responsibilities in terms of coordinating where and how their pets would be exercised. Other tenants were forced to live on the outskirts of the city, thus impacting how they scheduled their days so that their pets would not be left alone too long. Dogs can become destructive or disruptive when unexercised or when left alone too long, and the consequences could include eviction or relinquishment [3]. Tenants may leave sounds or lights on to keep their dogs company when left alone. Doing so, however, could become expensive, especially for those landlords and property managers who cover utilities. 

### 4.1. Implications for Research

A main suggestion that arose in our findings is for landlords and property managers to meet the pets of prospective tenants prior to signing the lease. Important to remember, however, is that the landlords and property managers who responded to our survey were all pet owners themselves. Such landlords and property managers may be more understanding of obstacles faced by pet owners in rental housing and perhaps more tolerant toward the behavior of pets once housed. By contrast, landlords and property managers who have limited experience with pet ownership may feel inadequately equipped to judge an animal’s appearance or their behavior prior to signing the lease. Future research should target landlords and property managers who are not pet owners themselves, to see what more can be learned. 

Results from our study suggest that restrictions on pets in rental housing may influence the types of dogs that younger adults acquire, to the extent that some may opt for smaller-sized dogs or for breeds commonly advertised as “good apartment dogs.” Another potential area of research could be to look at why tenants acquire the dogs that they do, and whether housing circumstances influence this decision. Conducting such research may help to explain whether the rental market contributes to the popularity of dogs who suffer from welfare problems [21], given that smaller-sized dogs, including Bulldogs and Pugs, are regularly marketed as ideal companions for apartment living [22]. 

A final implication for future research could be to test for discrimination against pet owners in rental housing by conducting a field experiment similar to audit designs (see correspondence tests: [23,24,25,26,27,28,29]). Specifically, written inquiries could be sent to landlords and property managers to investigate how the probability of receiving a positive answer is affected by: (i) tenants’ characteristics (e.g., age); (ii) tenants’ dogs’ characteristics (e.g., number, size, breed); (iii) the offer to pay a pet fee and its amount, regardless of whether that was mentioned in the listing; (iv) the offer to send pictures of dogs, to send pet references, or to meet prior to signing of the lease; (v) the rental properties’ characteristics (e.g., type of housing, furnished, monthly rent, neighborhood type); and (vi) landlords’ or property managers’ characteristics (i.e., whether they are pet owners themselves, whether they live in the same building or home as their tenants with pets). Conducting such an experiment would allow researchers to test the results of our study.

### 4.2. Implications for Policy

Legislation should be considered in such a way that allows tenants with pets to attain quality rental housing without financial exploit. In our study, dog owners reported feeling discriminated against. An alternative explanation for this discrimination could be that landlords and property managers perceive younger tenants, regardless of whether they keep pets, as less economically stable than older tenants. Working under this assumption, landlords and property managers may be using pet-related surcharges to screen younger adults with dogs, whereby those who agree to pay extra show further economic stability and are thus seen as worthy of tenancy.

Yet animals may be relinquished when their owners are unable to afford additional pet fees required to access rental housing [30]. In our study, landlords and property managers suggested steering clear from furnished units or at the very least, recognizing the increased potential for pet-related damages when such units were rented out. Extra fees for pet ownership may thus only be needed for furnished properties since those were the rare instances in which pet-related damages were reported. In any case, previous research from our team shows that pet owners tend to search for non-furnished units as compared to furnished ones [8]. 

In the Canadian province of Alberta, where this study took place, landlords and property managers cannot legally ask for greater than one month’s rent as a general-purpose security deposit [17]. What this means, however, is that landlords and property managers must make any pet-related surcharges non-refundable since these are imposed in addition to the security deposit. In our study, the fact that pet fees were made non-refundable became a major reason for why younger tenants stayed put, as they did not want to lose more money by having to pay another pet fee they would never get back, should they move into another rental. If additional pet fees must be imposed, the amount should be a percentage of total monthly rent and should be capped. Additionally, the amount should be made refundable, to incentivize responsible pet ownership. 

We also note that in other jurisdictions, including some Canadian provinces, general-purpose security deposits and pet-related surcharges are not supposed to be levied. Further research on how tenants, landlords, and property managers negotiate such rules could be insightful. 

### 4.3. Implications for Practice

Greater collaboration between animal professionals and housing authorities could help reduce the risk of relinquishments. For instance, reputable dog trainers or animal behaviorists could help with meeting and evaluating pets prior to signing the lease. Shelters could also provide help by serving as resource hubs for issues related to pets in rental housing. In fact, the Calgary Humane Society already runs a behavior helpline for people who have recently adopted an animal [31]. This behavior helpline could be extended for tenants with pets who may be worried about eviction or relinquishment. 

Another strategy could be for landlords and property managers to partner with services of interest to tenants with pets, such as pet sitting, dog walking, dog training, grooming, or even pet-specific housing cleaning services. Upon signing the lease, landlords and property managers could highlight these services of interest. Also, they may wish to bring a copy of their city’s policies on pets. For instance, the City of Calgary has a Responsible Pet Ownership Bylaw, which recommends preventing pets from becoming a threat or nuisance [16].

An added suggestion that emerged in our findings was for landlords and property managers to ask for pet references. Animal professionals, including veterinarians, dog trainers, or animal behaviorists, could sign a standard form to help demonstrate that prospective tenants have access to proper supports in case their pet gets sick or shows any behavioral issues throughout the length of the tenancy. A pet policy, which lists the number and types pets permitted in the property and which includes clauses related to damage and cleaning, is also advisable.

Finally, qualifications to adopt a pet should be considered in such a way that does not discriminate against those who cannot necessarily afford homeownership. Often, shelters and rescue organizations require aspiring adopters to have a fenced-in yard or for already tenants to provide written consent from their landlords or property managers. These stringent qualifications may dissuade some people from adopting. In particular, the lengthy process of getting pets approved by condominium boards impacts people’s capacities to adopt. By the time dogs of interest may be approved, they may be placed elsewhere. In our study, the easiest way for younger tenants to acquire a dog was not through a reputable breeder or rescue organization, but through online classified sites, thus impacting the ways in which pets are sourced and potentially increasing behavioral issues or veterinary costs in the future.

## 5. Conclusions

The rhetoric of many shelters and animal control facilities is that “a pet is a commitment for life,” yet this message is in stark contrast with animals being advertised as merely negotiable by landlords and property managers in their listings for rental housing. By comparing the perspectives of landlords and property managers with those of younger tenants with dogs, our aim was to seek areas of possible convergence. The suggestions provided by landlords and property managers were supported by younger tenants with dogs, thus illuminating possibilities for negotiating differences when it comes to pets in rental housing.
